# Sex differences in the effects of mental fatigue on single-leg drop landing biomechanics among sport science university students

**DOI:** 10.3389/fpsyg.2025.1678338

**Published:** 2025-10-06

**Authors:** Zilong Wang, Ziqi Feng, Huizi Cui, Lingyue Meng, Pengfei Wang, Mengya Lu, Tao Liu, Qiuxia Zhang, Xiangdong Wang

**Affiliations:** 1 School of Physical Education, Jimei University, Xiamen, China; 2 School of Public Foundation, Dalian University of Technology, Panjin, China; 3 School of Physical Education, Soochow University, Suzhou, China; 4 Department of Sports and Leisure, Dongshin University, Naju, Jeollanam-do, Republic of Korea

**Keywords:** mental fatigue, single-leg drop landing, sex differences, biomechanics, sports injury

## Abstract

**Objective:**

To investigate the sex-differentiated effects of Mental Fatigue (MF) on lower extremity biomechanical characteristics during single-leg drop landing among sport science university students.

**Methods:**

Twenty-eight healthy sport science university students (14 females, 14 males) performed single-leg drop landings from a 30 cm height before and after MF induction via a 45-min Stroop task. Kinematic and kinetic data during landing were synchronously captured using a Vicon infrared motion capture system and Kistler force plates. A 2 × 2 mixed-design analysis of variance (ANOVA) was employed.

**Results:**

At Initial Contact (IC), males exhibited significantly smaller ankle plantarflexion angles post-MF compared to baseline (*p* < 0.001), and also significantly smaller than females post-MF (*p* = 0.005). Post-MF, females exhibited significantly smaller knee flexion angles than males (*p* = 0.004). For ankle inversion angle, only a significant main effect of sex was observed (*p* = 0.004). No significant differences were found for hip angles or kinetic variables at peak vGRF.

**Conclusion:**

At IC, males compensated by reducing ankle plantarflexion, while females compensated by reducing knee flexion, indicating that MF induces a sex-specific strategic reorganization of distal-proximal joint control. These differences disappeared during the peak loading phase, suggesting that MF primarily affects early anticipatory mechanisms rather than the entire impact absorption process. Ankle inversion angle showed only a sex main effect, and hip strategy remained unchanged, further highlighting a hierarchical control logic prioritizing “ankle-knee first, hip later.” This study not only validates the existence of an MF × sex interaction effect but also underscores the theoretical value of non-significant indicators under the null hypothesis.

## 1 Introduction

Within contemporary competitive sports and rehabilitation medicine, cognitive load has evolved from an implicit factor to a core training variable (Delgado et al., 2025; Leone et al., 2017; Wang et al., 2024a). Decision-making pressures during high-intensity sports, such as tactical execution and environmental anticipation, expose athletes to persistent cognitive demands, potentially leading to cumulative cognitive load (Mariano et al., 2023). This sustained cognitive demand may impair sensorimotor integration efficiency by competing for finite neural resources, consequently affecting reaction speed and movement execution capability (Schampheleer and Roelands, 2024; Staiano et al., 2024). In this context, Mental Fatigue (MF), a typical state of cognitive resource depletion, may constitute a significant factor influencing athletic performance (Kong et al., 2023).

MF is a subjective state of fatigue induced by prolonged cognitive tasks, characterized by diminished attention, delayed decision-making, and heightened perception of effort (Marcora et al., 2009). Cognitive neuroscience research indicates that MF can inhibit prefrontal cortex activity, impair neuromuscular control, and lead to reduced movement economy and aberrant movement patterns (Soutschek and Tobler, 2020). Notably, this cognitive load extends beyond athletic contexts and is pervasive in the structured cognitive demands of modern life, particularly with the ubiquity of technology and accelerated lifestyles, where individuals face continuous multitasking and cognitive resource allocation challenges. For instance, frequent smartphone use, sleep deprivation, and multitasking can all trigger MF, and this fatigued state is difficult to fully recover from within short timeframes (Filipas et al., 2021; Thompson et al., 2019; Thompson et al., 2020). To simulate this complex cognitive environment experimentally, researchers widely employ paradigms such as Stroop, n-back, Go/No-Go, and Flanker tasks, providing standardized interventions for studying MF’s impact on motor control (Arenales Arauz et al., 2024; Zhao and Jia, 2019; Lopes et al., 2020; Vrijkotte et al., 2018). However, while existing research has examined MF’s effect on sports performance, the biomechanical implications for dynamic, high-impact movements like landing remain unclear.

The single-leg drop landing is a critical technical component in high-risk sports such as basketball and volleyball (Wang et al., 2024b). Instability in joint posture at IC and abnormal impact loading have been strongly correlated with non-contact injuries like ACL rupture and ankle sprain (Belcher et al., 2024; Jeon et al., 2025). In real sports scenarios, this association is complicated by cognitive load, particularly concerning sensorimotor integration within movement control strategies (Jeon et al., 2025). Athletes must execute technical skills under cognitive load, and MF may alter their movement strategies by disrupting sensorimotor integration (Kong et al., 2023). However, it remains unclear whether sex differences in neuromuscular control persist under MF, and whether males and females exhibit divergent adaptive strategies in such cognitively demanding conditions. Indeed, sex differences, stemming from variations in anatomy, hormonal levels, and neuromuscular recruitment strategies, may modulate joint loading and stability during landing (Alizadeh et al., 2023). For example, females exhibit higher motor unit discharge rates and variability in plantar flexors, knee extensors, and tibialis anterior, while males possess greater muscle strength and stiffness (Guo et al., 2022), potentially leading to differential tolerance to MF. Although sex specificity and its interaction with MF have received some attention, research specifically examining sex differences in lower extremity biomechanics during single-leg drop landing before and after MF induction remains scarce. However, the extant literature has predominantly focused on reporting statistically significant outcomes, often overlooking the theoretical and practical value of non-significant findings. This selective reporting can paint an incomplete picture of MF’s effects. As Abadie (2020) argued, results consistent with the null hypothesis are foundational for theoretical revision and should be explicitly reported and discussed. Therefore, to gain a comprehensive understanding of the sex-specific impact of MF on landing biomechanics—encompassing both the presence and absence of effects—this study will meticulously analyze and interpret both significant and non-significant indicators. This approach is crucial for moving beyond a simplistic binary interpretation of MF’s effects and for providing the nuanced evidence base needed to develop truly personalized fatigue monitoring and injury prevention strategies. This not only facilitates a deeper understanding of sex-specific landing biomechanics but also provides crucial references for developing personalized fatigue recovery training protocols in sports performance.

Accordingly, this study employed the classic Stroop task to induce MF, investigating its differential impact on lower extremity biomechanics during single-leg drop landing between sexes. By comparing the response differences in joint kinematics and kinetics between healthy males and females, the factors influencing MF on landing motor control across sexes were elucidated. Both significantly and non-significantly different indicators were reported and discussed to provide an important reference for developing sex-specific fatigue training programs in sports performance. Based on prior research, the following hypotheses were proposed: (1) Under MF, sex differences will be observed in ankle and knee joint angles in the sagittal plane at initial contact during single-leg drop landing. (2) MF will induce sex-specific compensatory strategies, with males reducing ankle plantarflexion and females reducing knee flexion at initial contact.

## 2 Methods

### 2.1 Participants

Using G*Power 3.1.9 software, with reference to previous studies and the statistical methods employed herein (Ma et al., 2025; Romagnoli et al., 2024), the required sample size was estimated. Power (1-*β*), type I error (*α*), and effect size (f) were set to 0.80, 0.05, and 0.40, respectively, yielding a minimum sample size of 16 participants. Ultimately, twenty-eight healthy sport science university students (14 males, 14 females) were recruited. The male group included 5 students specializing in basketball, 4 in volleyball, 3 in badminton, and 2 in tennis. The female group had an identical composition. The dominant leg was determined using the kick-ball test (Ren et al., 2021). All assessments and screenings were conducted by an experienced experimenter. Prior to the experiment, all participants were informed of the testing procedures and provided written informed consent. The study was approved by the Ethics Committee of Soochow University (Ethics Approval No.: SUDA20250425H04). Participant Characteristics are presented in Table 1.

**Table 1:** Participant characteristics (M ± SD).

Basic information	Males (*n* = 14)	Females (*n* = 14)
Age (years)	21.9 ± 1.1	22.3 ± 1.2
Height (cm)	177.0 ± 6.3*	165.7 ± 3.4*
BMI (kg/m^2^)	21.7 ± 2.3	21.0 ± 1.7
weight (kg)	75.9 ± 8.4	57.6 ± 4.4
Tegner Activity Score	5.2 ± 1.1	5.1 ± 1.2
Dominant Side (Left/Right)	3/11	0/14

*p < 0.05.

Inclusion criteria: (1) No history of lower limb joint or neuromuscular diseases or injuries within the past six months; (2) Similar morphological characteristics and optimal physical fitness level; (3) No strenuous exercise within 24 h prior to testing and avoidance of caffeine intake; (4) Avoidance of alcohol consumption for ≥1 week prior to testing; (5) Good psychological state, absence of severe psychological disorders or mental stress; (6) Exercise frequency ≥3 times per week; (7) Female participants not menstruating on the test day.

### 2.2 Instrumentation

#### 2.2.1 Vicon infrared 3D motion capture system

The system comprised 8 infrared cameras (Model: MX13, UK), MX Net, MX Control, a PC workstation, a calibration kit, and standard accessories. Sampling frequency was set at 100 Hz, which is consistent with previous studies investigating drop landing kinematics and provides sufficient temporal resolution to capture the primary joint angle changes during the landing phase (Ren et al., 2021). The CGM 2.3 lower limb model within the Vicon system was utilized.

#### 2.2.2 Three-dimensional force plates

Two Kistler three-dimensional force plates (Dimensions: 90 cm × 60 cm × 10 cm; Model: 9287B, Switzerland) were embedded in the floor at the center of the motion capture system’s camera range. Sampling frequency was 1,000 Hz. An analog-to-digital converter synchronized the Kistler force plates with the Vicon motion capture system for data acquisition.

### 2.3 Experimental design and procedures

**Figure 1:**
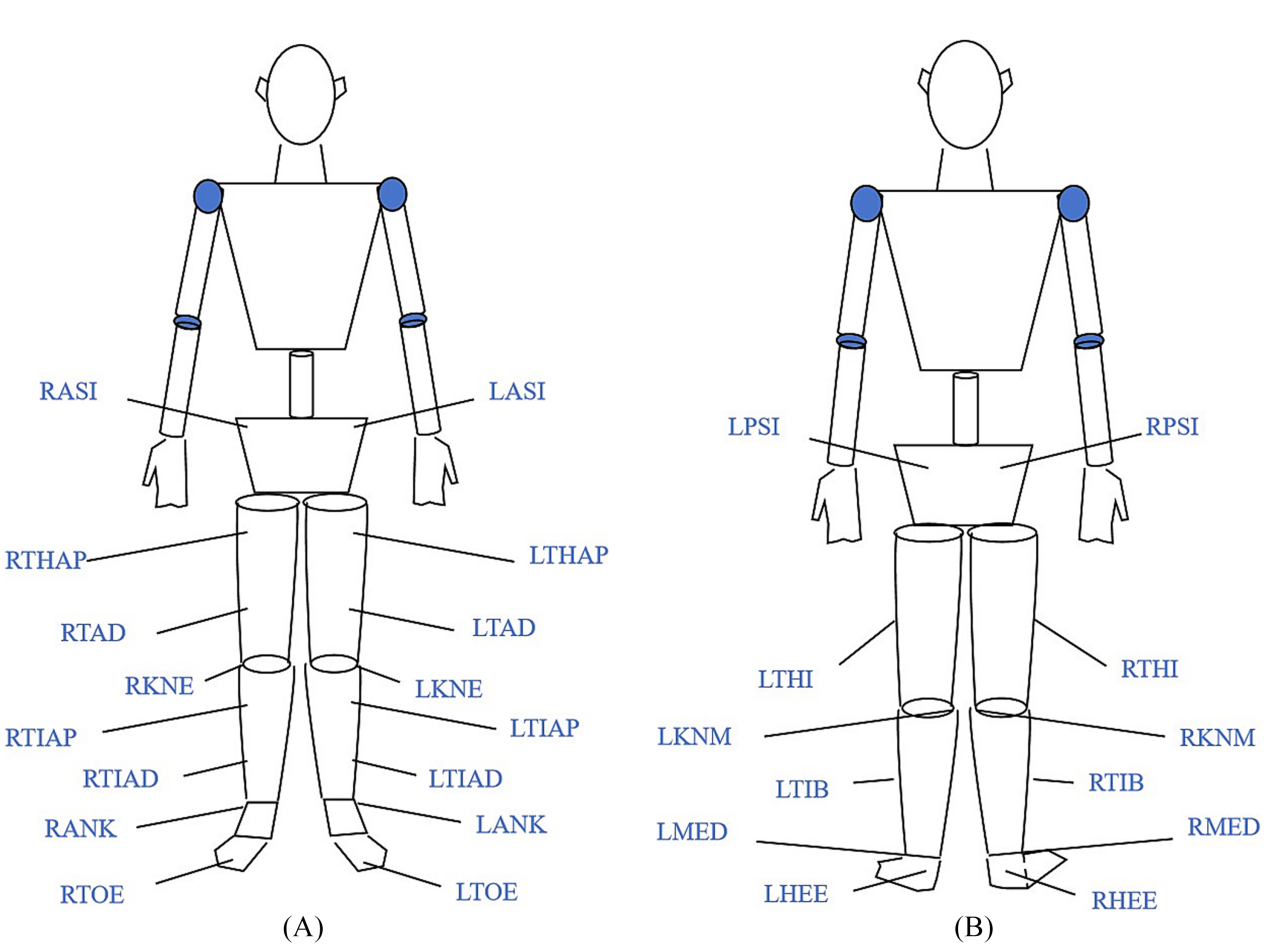
Marker point pasting **(A)** Front view; **(B)** Back view. Adapted from Wang et al. (2024b). LANK: Left lateral malleolus; LASI: Left anterior superior iliac spine; LHEE: Left heel; LKNM: Left medial knee; LKNE: Left lateral knee; LMED: Left medial malleolus; LPSI: Left posterior superior iliac spine; LTHAP: Left thigh proximal; LTHI: Left thigh; LTAD: Left thigh distal; LTIB: Left tibia; LTIAD: Left tibia distal; LTIAP: Left tibia proximal; LTOE: Left toe; RANK: Right lateral malleolus; RASI: Right anterior superior iliac spine; RHEE: Right heel; RKNM: Right medial knee; RKNE: Right lateral knee; RMED: Right medial malleolus; RPSI: Right posterior superior iliac spine; RTHAP: Right thigh proximal; RTHI: Right thigh; RTAD: Right thigh distal; RTIB: Right tibia; RTIAD: Right tibia distal; RTIAP: Right tibia proximal; RTOE: Right toe.

All participants performed the single-leg drop landing task before and after MF induction. Participants were instructed to avoid caffeine intake and strenuous exercise for 24 h prior to each test session and to maintain regular sleep patterns. Before each experiment, participants wore standardized tight-fitting shorts and athletic shoes provided by the laboratory. The 10-min warm-up protocol consisted of: (1) 3 min treadmill jogging at 6 km/h, 0% incline; (2) 3 min dynamic lower-limb activation (5 forward/backward and 5 lateral leg swings, 5 hip circles each leg); (3) 2 min ankle-specific mobilization (ankle pumps, alphabet tracing, and PNF diagonals, 30 s each); (4) 2 min sub-maximal vertical hops (3 sets of 3 hops at ~50% perceived effort). Following a warm-up, 28 retroreflective markers (14 mm diameter) were affixed to corresponding anatomical landmarks according to the CGM 2.3 lower limb model protocol (Figure 1) (Wang et al., 2024b). To ensure accuracy, all marker placements were performed by the same experienced technician.

### 2.4 Experimental protocol

#### 2.4.1 MF protocol

Given substantial prior evidence confirming that the Stroop task reliably induces MF within 30–60 min (Cuchna et al., 2024; Faria et al., 2024; Wu et al., 2025; Romagnoli et al., 2024), and to avoid prolonging the experimental period with washout phases for a passive control group (e.g., watching videos) which could introduce confounding variables, no passive control condition was implemented. The MF intervention consisted of a continuous 45-min computerized Stroop task, designed based on studies by Lopes et al. (2020) and Wang et al. (2025), and pre-testing results. This method has been validated for effective MF induction (Mangin et al., 2022). The task procedure was as follows: The Chinese characters for “red,” “green,” “blue,” and “yellow” were displayed sequentially in random order on a computer screen. Each character was displayed in one of the four colors, with a 50% probability of incongruence between word meaning and font color. Participants were required to press a key corresponding to the font color. Each character was displayed for 1,000 ms, followed by a 1,000 ms blank screen, totaling 1,350 judgments. Participants were instructed to respond as quickly and accurately as possible. An auditory cue sounded for incorrect responses or failures to respond within 1,500 ms to encourage greater focus. The experiment was conducted in a quiet, isolated room using E-Prime 3.0 software. Two experimenters supervised the session to ensure protocol adherence and participant focus. Considering the Stroop task’s potential to affect participants multi-dimensionally, the NASA-TLX (National Aeronautics and Space Administration Task Load Index) subjective workload scale was employed to precisely assess MF status and associated perceptual changes (Said et al., 2020; Dias et al., 2018). Participants remained seated throughout, with no additional physical exertion, and the NASA-TLX results further confirmed that fatigue originated solely from cognitive demand. To address potential ambiguity in university students’ perception of MF, a clear, standardized definition of MF was provided during the familiarization phase, along with instructions for self-assessment, to minimize bias in self-reporting.

#### 2.4.2 Single-leg drop landing protocol

**Figure 2:**
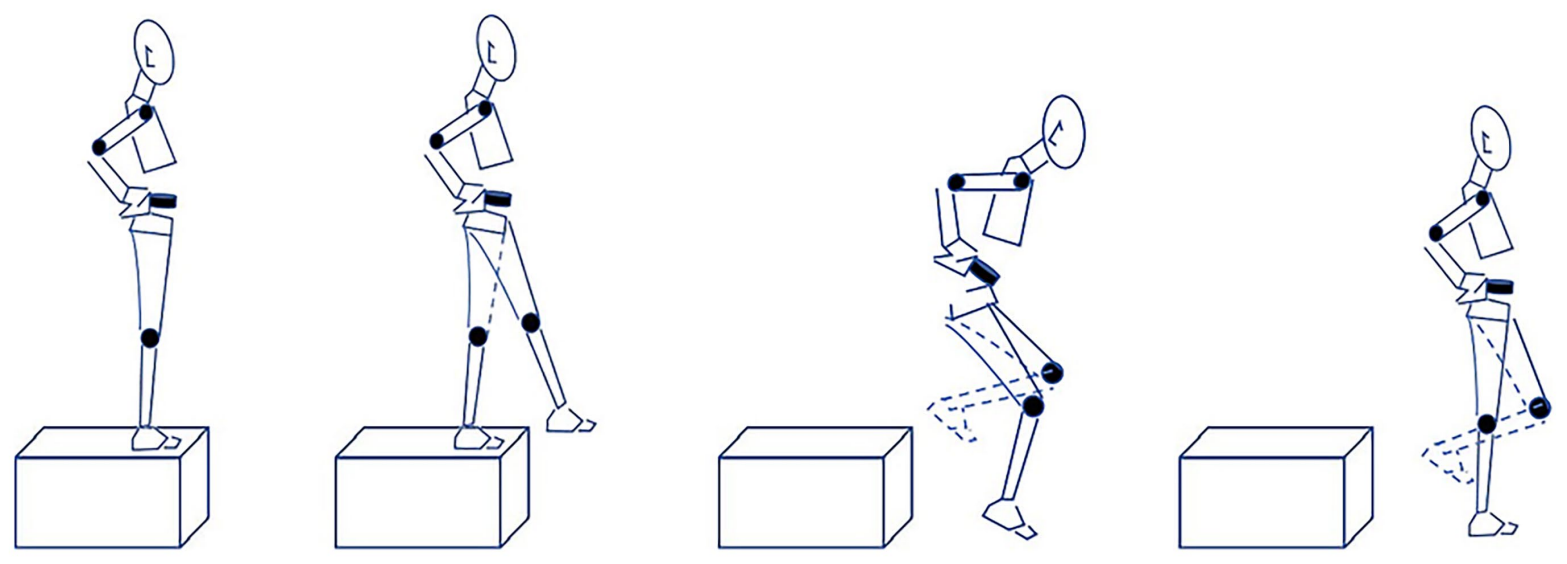
Schematic of single-leg drop landing task.

The single-leg drop landing test was designed based on previous studies by Meng et al. (2022) and Wang et al. (2024b). Participants stood naturally on a 30 cm high box with feet shoulder-width apart. During testing, hands were placed on the hips to avoid inertial effects from arm swing. Participants were instructed to perform a single-leg landing onto the force plates using a “toe-heel” landing pattern (Figure 2). Prior to formal testing, all participants were given sufficient practice trials to familiarize themselves with and master the landing maneuver. To reduce random error and enhance data reliability, participants performed three successful trials for their dominant leg both pre- and post-MF. The average of the three trials was used for analysis.

### 2.5 Data processing and analysis

Raw data captured by the Vicon system were processed for kinematic and inverse dynamic analysis using Visual3D software. The cut-off frequencies for filtering (10 Hz for kinematics, 50 Hz for kinetics) were selected based on established practices in biomechanics literature to effectively remove noise while preserving the true biological signal characteristics of these data types. Lower limb joint angles were calculated using the Euler angle method. IC was defined as the instant when the vertical Ground Reaction Force (vGRF) first exceeded 10 N (Wang et al., 2024a).

The primary outcome measures were: (1) Hip, knee, and ankle joint angles (°) in the sagittal and frontal planes at IC and at the time of peak vGRF. (2) Peak vGRF normalized to body weight (BW). (3) Time to peak vGRF (T_vGRF) (ms). (4) Vertical loading rate (LR) (BW/ms). (5) Change in lower limb length (ΔL) (m), calculated as the difference between limb length at initial contact and the minimum limb length during the landing phase. Limb length was defined as the vertical distance from the greater trochanter marker to the ground. (6) Leg stiffness ( $K_{\mathrm{leg}}$ ) (BW/m), calculated as the peak vGRF divided by ΔL (Meng et al., 2022). LR and $K_{\mathrm{leg}}$ were calculated using Equations (1, 2) respectively:


LR=peak vGRF/T_vGRF (1)

$$K_{\mathrm{leg}}=\text{peak vGRF}/\mathrm{\Delta L}$$ (2)

### 2.6 Statistical analysis

Data were analyzed using SPSS 27.0 software. Descriptive statistics are presented as mean ± standard deviation (M ± SD). The Shapiro–Wilk test assessed normality, and Levene’s test assessed homogeneity of variance. A 2 (Sex: Male, Female) × 2 (Condition: MF, Baseline) mixed-design repeated-measures ANOVA was used to analyze kinematic and kinetic data. Statistical significance was set at *α* = 0.05. Upon significant interaction effects, simple effects analysis was performed, with the significance level adjusted to *p* < 0.0125 to account for four primary comparisons.

## 3 Results

### 3.1 MF intervention efficacy

**Figure 3:**
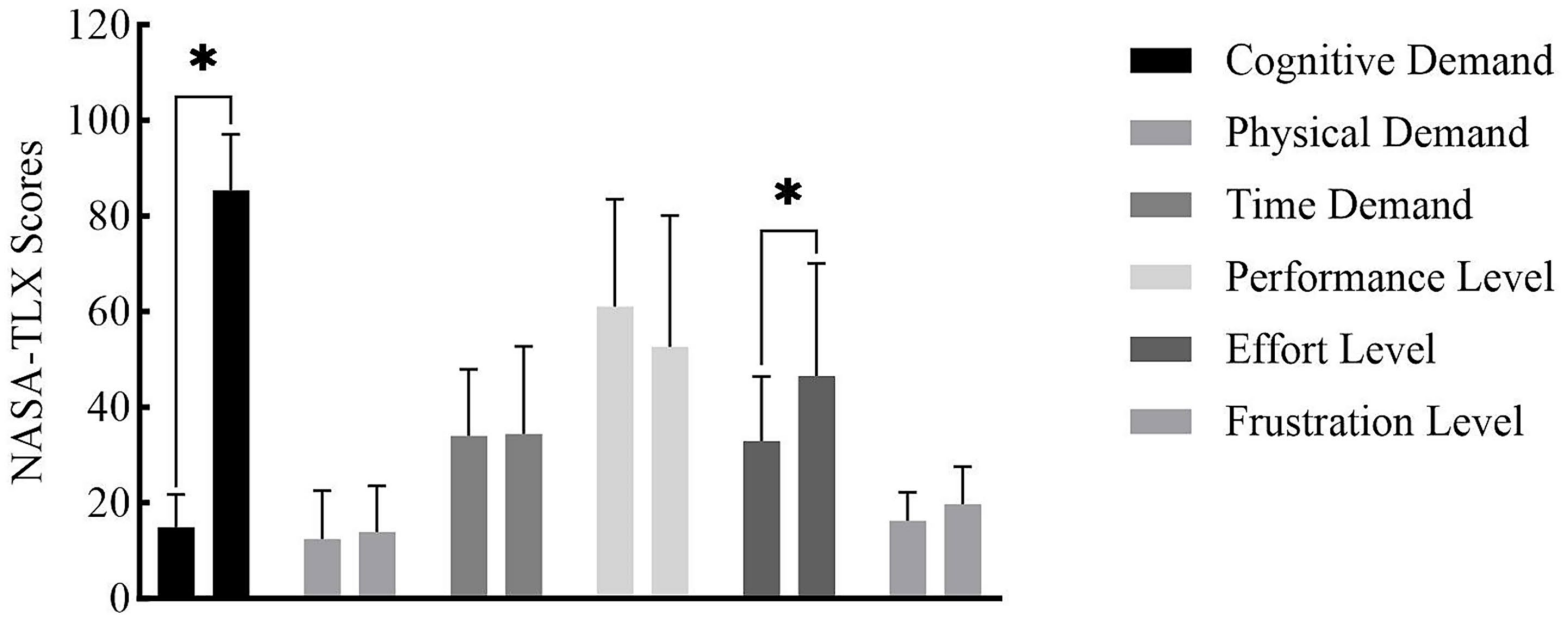
NASA-TLX scores before and after MF induction.

Significant increases were observed in the NASA-TLX subscales of Cognitive Demand (*p* < 0.001) and Effort Level (*p* = 0.027) post-MF, confirming successful MF induction. No significant differences (*p* > 0.05) were found for Physical Demand, Time Demand, Performance Level, or Frustration Level, indicating the Stroop task did not significantly impact these dimensions (Figure 3).

### 3.2 Hip, knee, and ankle joint angles at IC

A significant MF × Sex interaction was observed for ankle plantarflexion angle at IC (*p* < 0.001). Simple effects analysis revealed that males exhibited significantly smaller ankle plantarflexion angles post-MF compared to baseline (*p* < 0.001) and also compared to females post-MF (*p* = 0.005). For ankle inversion angle at IC, a significant main effect of sex was found (*p* = 0.004), with males exhibiting smaller inversion angles than females. A significant MF × Sex interaction was observed for knee flexion angle at IC (*p* = 0.008). Simple effects analysis showed that females exhibited significantly smaller knee flexion angles than males post-MF (*p* = 0.004). No significant differences were found for the remaining variables at IC (*p* > 0.05) (Table 2).

**Table 2:** Hip, knee, and ankle joint angles at IC during single-leg drop landing.

Variable	Male	Female	Main effect		Interaction effect
			MF	Sex	MF × Sex
Hip Flexion (+)/Extension (−) Angle (°)					
Baseline	19.54 ± 6.13	24.48 ± 3.95	*p* = 0.318	*p* = 0.083	*p* = 0.300
			*F* = 1.036	*F* = 3.255	*F* = 1.118
MF	19.59 ± 7.19	22.12 ± 7.15	*Eta^2^* = 0.038	*Eta^2^* = 0.111	*Eta^2^* = 0.041
Hip Adduction (+)/Abduction (−) Angle (°)					
Baseline	−6.03 ± 3.20	−4.62 ± 3.03	*p* = 0.418	*p* = 0.279	*p* = 0.595
			*F* = 0.678	*F* = 1.221	*F* = 0.289
MF	−5.03 ± 2.28	−4.41 ± 3.77	*Eta^2^* = 0.025	*Eta^2^* = 0.045	*Eta^2^* = 0.011
Knee Flexion (+)/Extension (−) Angle (°)					
Baseline	8.40 ± 3.74	8.79 ± 4.10	*p* = 0.463	*p* = 0.197	***p* = 0.008** ^*^
			*F* = 0.554	*F* = 1.757	*F* = 8.231
MF	9.79 ± 2.89	6.43 ± 2.83	*Eta^2^* = 0.021	*Eta^2^* = 0.063	*Eta^2^* = 0.240
Knee Varus (+)/Valgus (−) Angle (°)					
Baseline	1.45 ± 2.39	0.76 ± 2.34	*p* = 0.760	*p* = 0.490	*p* = 0.790
			*F* = 0.095	*F* = 0.491	*F* = 0.073
MF	1.00 ± 3.00	0.73 ± 3.13	*Eta^2^* = 0.004	*Eta^2^* = 0.019	*Eta^2^* = 0.003
Ankle Dorsiflexion (+)/Plantarflexion (−) Angle (°)					
Baseline	−21.14 ± 4.20	−21.17 ± 4.05	***p* = 0.007** ^*^	*p* = 0.093	***P* < 0.001** ^*^
			*F* = 8.651	*F* = 3.046	*F* = 16.759
MF	−16.60 ± 4.56	−21.91 ± 4.71	*Eta^2^* = 0.250	*Eta^2^* = 0.105	*Eta^2^* = 0.392
Ankle Inversion (+)/Eversion (−) Angle (°)					
Baseline	2.10 ± 2.34	3.40 ± 1.68	*p* = 0.570	***p* = 0.004** ^*^	*p* = 0.552
			*F* = 0.332	*F* = 9.733	*F* = 0.363
MF	1.43 ± 1.87	3.41 ± 2.22	*Eta^2^* = 0.013	*Eta^2^* = 0.272	*Eta^2^* = 0.014

Bold values indicate statistically significant differences.

### 3.3 Hip, knee, and ankle joint angles at peak vGRF

No statistically significant differences were observed for any hip, knee, or ankle joint angle at the time of peak vGRF (*p* > 0.05) (Table 3).

**Table 3:** Hip, knee, and ankle joint angles at peak vGRF during single-leg drop landing.

Variable	Male	Female	Main effect		Interaction effect
			MF	Sex	MF × Sex
Hip Flexion (+)/Extension (−) Angle (°)					
Baseline	23.85 ± 9.15	28.27 ± 4.21	*p* = 0.172	*p* = 0.236	*p* = 0.324
			*F* = 1.974	*F* = 1.469	*F* = 1.009
MF	23.37 ± 8.43	25.40 ± 8.09	*Eta^2^* = 0.071	*Eta^2^* = 0.053	*Eta^2^* = 0.037
Hip Adduction (+)/Abduction (−) Angle (°)					
Baseline	−5.32 ± 3.65	−3.51 ± 3.06	*p* = 0.528	*p* = 0.547	*p* = 0.205
			*F* = 0.409	*F* = 0.373	*F* = 1.687
MF	−3.49 ± 3.75	−4.13 ± 3.78	*Eta^2^* = 0.015	*Eta^2^* = 0.014	*Eta^2^* = 0.061
Knee Flexion (+)/Extension (−) Angle (°)					
Baseline	27.40 ± 4.97	26.45 ± 6.44	*p* = 0.149	*p* = 0.274	*p* = 0.281
			*F* = 2.216	*F* = 1.250	*F* = 1.212
MF	27.04 ± 3.03	24.04 ± 6.04	*Eta^2^* = 0.079	*Eta^2^* = 0.046	*Eta^2^* = 0.045
Knee Varus (+)/Valgus (−) Angle (°)					
Baseline	0.77 ± 4.33	0.33 ± 4.70	*p* = 0.997	*p* = 0.898	*p* = 0.837
			*F* < 0.001	*F* = 0.017	*F* = 0.043
MF	0.63 ± 4.81	0.47 ± 5.13	*Eta^2^* < 0.001	*Eta^2^* = 0.001	*Eta^2^* = 0.002
Ankle Dorsiflexion (+)/Plantarflexion (−) Angle (°)					
Baseline	13.49 ± 3.62	13.08 ± 3.99	*p* = 0.616	*p* = 0.379	*p* = 0.376
			*F* = 0.257	*F* = 0.800	*F* = 0.810
MF	14.76 ± 5.67	12.73 ± 3.61	*Eta^2^* = 0.010	*Eta^2^* = 0.030	*Eta^2^* = 0.030
Ankle Inversion (+)/Eversion (−) Angle (°)					
Baseline	2.32 ± 2.57	0.98 ± 3.48	*p* = 0.324	*p* = 0.378	*p* = 0.533
			*F* = 1.009	*F* = 0.804	*F* = 0.399
MF	1.38 ± 3.46	0.76 ± 3.47	*Eta^2^* = 0.037	*Eta^2^* = 0.030	*Eta^2^* = 0.015

### 3.4 Peak vGRF, T_vGRF, ΔL, Kleg, and LR before and after MF

No statistically significant differences were observed for peak vGRF, T_vGRF, ΔL, Kleg, or LR (*p* > 0.05) (Table 4).

**Table 4:** Peak vGRF, T_vGRF, LR, ΔL, and K_leg_ during single-leg drop landing.

Variable	Male	Female	Main effect		Interaction effect
			MF	Sex	MF
peak vGRF (BW)					
Baseline	2.58 ± 0.56	2.75 ± 0.55	*p* = 0.390	*p* = 0.748	*p* = 0.079
			*F* = 0.764	*F* = 0.105	*F* = 3.333
MF	2.91 ± 0.54	2.63 ± 0.54	*Eta^2^* = 0.029	*Eta^2^* = 0.004	*Eta^2^* = 0.114
T_vGRF (ms)					
Baseline	84.29 ± 34.35	70.00 ± 17.10	*p* = 0.103	*p* = 0.432	*p* = 0.103
			*F* = 2.862	*F* = 0.636	*F* = 2.862
MF	67.14 ± 18.16	70.00 ± 18.81	*Eta^2^* = 0.099	*Eta^2^* = 0.024	*Eta^2^* = 0.099
ΔL (m)					
Baseline	0.13 ± 0.03	0.12 ± 0.02	*p* = 0.760	*p* = 0.099	*p* = 0.816
			*F* = 0.095	*F* = 2.929	*F* = 0.055
MF	0.14 ± 0.03	0.12 ± 0.02	*Eta^2^* = 0.004	*Eta^2^* = 0.101	*Eta^2^* = 0.002
K_leg_ (BW/m)					
Baseline	20.32 ± 6.11	23.43 ± 2.99	*p* = 0.428	*p* = 0.435	*p* = 0.133
			*F* = 0.647	*F* = 0.629	*F* = 2.407
MF	22.88 ± 7.62	22.62 ± 4.32	*Eta^2^* = 0.024	*Eta^2^* = 0.024	*Eta^2^* = 0.085
LR (BW/ms)					
Baseline	0.35 ± 0.16	0.42 ± 0.16	*p* = 0.124	*p* = 0.911	*p* = 0.073
			*F* = 2.522	*F* = 0.013	*F* = 3.498
MF	0.47 ± 0.18	0.42 ± 0.18	*Eta^2^* = 0.088	*Eta^2^* < 0.001	*Eta^2^* = 0.119

## 4 Discussion

This study investigated the sex-differentiated effects of MF on lower extremity biomechanics during single-leg drop landing in a cohort of sport science students. It is important to note that the following findings and interpretations are primarily applicable to young, healthy, and physically active individuals with a structured background in physical activity and sports training, specifically focusing on kinematic and kinetic responses at IC and peak loading. The results revealed significant MF × Sex interactions for ankle plantarflexion and knee flexion angles at IC. This finding supports both hypotheses: MF induces sex-specific biomechanical responses during landing, with differences concentrated at IC. Males adopted a “stiffening” strategy by reducing ankle plantarflexion, while females reduced knee flexion, potentially increasing ACL injury risk, demonstrating divergent motor control logics within the critical initial time window. Specifically, males exhibited significantly smaller ankle plantarflexion angles post-MF compared to females and their own baseline, which could be interpreted as a preference for a ‘stiff’ landing strategy. It is worth noting that although participants were instructed to adopt a ‘toe-heel’ landing pattern, kinematic analysis confirmed that all trials consistently exhibited forefoot contact first, followed by heel strike. Nevertheless, the observed sex differences in ankle angles suggest that the instructed strategy was executed with subtle variations, possibly reflecting sex-specific neuromuscular adaptations to MF. This aligns with Zhang et al.'s (2021) findings on physical fatigue, where landing strategies shifted from “soft” to “stiff” with increasing fatigue, and the ankle’s role in impact absorption became more prominent, indicating fatigue drives individuals to increase distal joint stiffness to reduce load and energy cost. Our study further reveals that males exhibit a similar adaptation post-MF, this may suggest that reduced ankle plantarflexion serves as a compensatory strategy under fatigue, though further neurophysiological evidence is needed to confirm this mechanism. Conversely, females exhibited significantly smaller knee flexion angles than males post-MF, suggesting that the additional cognitive load from MF impaired their active buffering capacity, potentially increasing ACL loading risk. Withrow et al.'s (2006) seminal simulation study demonstrated a significant positive correlation between increased knee flexion angle and reduced ACL strain during landing, indicating greater knee flexion better dissipates impact forces. Conversely, smaller knee flexion angles lead to more direct force transmission to the ACL, increasing strain risk. This observed sex difference might reflect divergent neuromuscular adaptation strategies, males may maintain system stability by reducing joint range of motion under fatigue, while females may passively accept greater joint loads due to neural inhibition or reduced motor unit recruitment efficiency. Although significant at IC, these differences disappeared at peak loading, suggesting MF’s sex-differentiated effects primarily target the early “preparatory regulation” phase of landing rather than the mid-to-late impact absorption phase. This implies MF shapes sex-specific landing strategies by influencing central anticipatory mechanisms rather than peripheral execution.

Simultaneously, ankle inversion angle at IC exhibited only a significant main effect of sex, with males showing smaller inversion angles both pre- and post-MF. This difference likely stems from inherent anatomical or neuromuscular control strategy differences between sexes (Ahtiainen et al., 2003; Cadore et al., 2012; Mancino et al., 2024), with no significant interaction with MF. This finding aligns with Hunter's (2014)view that physiological and anatomical sex differences are key factors underlying neuromuscular performance disparities. Notably, hip angles at IC showed no significant main or interaction effects, suggesting hip posture regulation during initial landing may be influenced more by individual motor experience or task familiarity than by MF or sex factors. This result diverges from Jacobs et al. (2007) and Gehring et al. (2014), who emphasized the critical role of hip abductors in female motor control. This discrepancy may arise because their studies artificially amplified hip strategy demands through fatiguing hip abductor exercises or prolonged running loads; our study employed brief, anticipated single-leg drops without imposing additional load on hip abductors, causing sex differences to manifest primarily in ankle rather than hip strategy. This observed pattern of joint-specific adjustments, where significant MF × sex effects were found at the ankle and knee at IC but not at the hip or during peak loading, suggests a potential hierarchical control logic in managing MF, which aligns with the principle of economy in motor control (Hayashibe and Shimoda, 2014). However, the interpretation of these findings must be considered within the context of our methodological design. We employed a standardized ‘toe-heel’ landing strategy to control for technical variability and enhance the internal validity for isolating the effects of MF. While this approach is common in biomechanical studies, it may limit the ecological validity of our findings, as it potentially suppresses the natural variations in landing strategy that athletes might employ under cognitive load in real-world settings. This trade-off between internal and external validity is a key consideration. Our instruction might have attenuated some fatigue-induced adaptations, suggesting that the sex-specific compensatory strategies we observed at the ankle and knee could represent a conservative estimate of the true effect of MF. Future studies should aim to balance this trade-off by incorporating both standardized and natural landing tasks to provide a more comprehensive understanding of how MF influences biomechanics in both controlled and ecologically valid environments. Furthermore, although participants were instructed to adopt a ‘toe-heel’ landing pattern, kinematic analysis confirmed that all trials consistently exhibited forefoot contact first, followed by heel strike. Nevertheless, the observed sex differences in ankle angles suggest that the instructed strategy was executed with subtle variations, reflecting inherent sex-specific neuromuscular control strategies that persist even under a constrained task.

Further analysis of non-significant indicators, such as joint angles at peak vGRF, peak vGRF, T_vGRF, ΔL, Kleg, and LR, suggests that MF and sex factors have limited impact on the overall kinetic characteristics of impact absorption. While these non-significant results may reflect a true absence of effect, they could also be influenced by statistical power or task design, and should be interpreted with caution. Instead, they reveal that MF’s effect on motor control may be task-phase specific. Subtle compensatory strategies at IC may be sufficient to buffer subsequent impact forces, resulting in no significant differences in kinematics and kinetics at peak loading. This perspective aligns with Abadie (2020)'s emphasis on the theoretical value of non-significant results, suggesting future research should focus on the “temporal window” effect in motor control. Higo and Kuruma's (2021) findings support this, showing that MF primarily affected pre-landing strategies like trunk flexion in males performing single-leg landings from 30 cm, while physical fatigue significantly altered peripheral kinetics. Similarly, Shumski et al. (2023) reported that MF’s effect on peak vGRF varied by individual and task type, with no significant increase observed in healthy populations, consistent with our findings. In contrast, Zheng et al. (2025) found MF increased peak vGRF in male university students performing stop-jump and single-leg landing tasks, suggesting MF alters biomechanics in high-intensity tasks. The discrepancy between our findings (no kinetic changes) and those of Zheng et al. (2025) (increased peak vGRF under MF) can likely be attributed to critical methodological differences in task design and participant recruitment, which ultimately dictate the ecological validity and neuromuscular demand of the landing task. First, regarding task design, Zheng et al. employed a stop-jump task, which is inherently more dynamic, unpredictable, and imposes a higher cognitive and physical load than our standardized single-leg drop landing from a static position. The stop-jump requires rapid deceleration, change of direction, and explosive propulsion, engaging muscle groups and neural control mechanisms differently. This higher-intensity task may be more sensitive to the detrimental effects of MF on kinetic outcomes. Second, the drop height used by Zheng et al. (40 cm) was greater than ours (30 cm), resulting in higher impact forces and potentially increasing the task’s sensitivity to detect MF-induced alterations in landing mechanics. Finally, the participant profile differed: while our cohort consisted of sport science students from various disciplines, Zheng et al. specifically recruited athletes from ball sports (basketball, soccer, volleyball) involving frequent jumping and cutting. These athletes are likely highly trained in absorbing high-impact landings specific to their sports, which might interact differently with MF compared to our more generalized active population. Crucially, these non-significant indicators, combined with the significant differences observed at IC, form a pattern of selective modulation by MF. The central nervous system appears to prioritize adjustments to degrees of freedom sensitive to injury risk (like IC joint angles), while preserving parameters with limited impact on overall kinetics (like angles at peak load), achieving an optimal balance between energy efficiency and risk control. Furthermore, the significant height difference between male and female participants in this study represents a structural variable that could potentially influence impact kinetics by altering center of mass height, drop time, and ground reaction force lever arms. While the 30 cm drop height is widely used in laboratory settings, it may not fully replicate the impact forces encountered in sport-specific landings. Future studies should consider using higher drop heights or sport-specific landing tasks to enhance ecological validity. Future studies should better control for height in statistical models or experimental designs to more precisely parse MF × sex interaction effects.

These observed sex-specific compensatory strategies under MF carry significant practical implications for injury prevention and athletic training. The finding that males primarily reduce ankle plantarflexion suggests that their landing technique becomes less effective at dissipating forces distally upon MF. Consequently, prevention programs for male athletes could incorporate cognitive load simulations into plyometric and landing drills, specifically emphasizing the maintenance of ankle mobility and control under fatigued conditions. This might involve performing landing exercises after sport-specific decision-making tasks or Stroop protocols. Conversely, the reduced knee flexion observed in fatigued females highlights a potentially increased risk for ACL injuries due to a more extended knee position at initial contact. Thus, female-focused interventions should prioritize reinforcing optimal knee flexion kinematics during landing, even under mentally demanding scenarios. Neuromuscular training programs could integrate cognitive challenges (e.g., reacting to auditory or visual stimuli) into exercises designed to promote soft landings with greater knee flexion, such as depth jumps or single-leg stabilization drills. Furthermore, our results underscore the importance of monitoring MF states, particularly in female athletes or in sports with high cognitive demands, as a preventative measure. Simple subjective scales like the NASA-TLX could be used alongside training to identify individuals exhibiting heightened cognitive fatigue, prompting adjustments in training load or the implementation of targeted recovery strategies. By acknowledging these sex-differentiated responses to MF, coaches and sports scientists can develop more personalized and effective training regimens aimed at mitigating injury risk in both competitive and recreational settings.

This study has several limitations: (1) Participants were sport science students; generalizing findings to the general population or elite athletes requires caution. (2) The induction of MF was verified solely via subjective scales (NASA-TLX), lacking support from objective data such as behavioral performance during the Stroop task (e.g., reaction time, accuracy trajectory) or neurophysiological indices. (3) Surface electromyography (sEMG) was not recorded, limiting mechanistic interpretation of sex differences at the neuromuscular activation level. Additionally, the use of a large effect size (*f* = 0.40) in our *a priori* power analysis may have led to an underpowered study for detecting smaller effects, which should be considered when interpreting the null findings. Future research should integrate high-density EMG and EEG to monitor corticomuscular coupling in real-time and extend investigations to real-world sports scenarios to enhance external validity.

## 5 Conclusion

This study investigating the sex-differentiated effects of MF on lower extremity biomechanics during single-leg drop landing found that at IC, males compensated by reducing ankle plantarflexion, while females compensated by reducing knee flexion. This indicates that MF induces a sex-specific strategic reorganization of distal-proximal joint control. These differences disappeared during the peak loading phase, demonstrating that MF primarily affects early anticipatory mechanisms rather than the entire impact absorption process. Ankle inversion angle exhibited only a sex main effect, and hip strategy remained unchanged, further highlighting a hierarchical control logic prioritizing “ankle-knee first, hip later.” This research not only validates the existence of an MF × sex interaction effect but also underscores the theoretical value of non-significant indicators under the null hypothesis. Future studies should employ larger samples, varying impact intensities, and real-world competitive settings, incorporating EMG–EEG synchrony techniques to elucidate sex-differentiated central-peripheral coupling pathways and provide precise targets for personalized fatigue monitoring and injury prevention interventions.

## Data Availability

The original contributions presented in the study are included in the article/supplementary material, further inquiries can be directed to the corresponding authors.
