# Correction: Sex differences in the effects of mental fatigue on single-leg drop landing biomechanics among sport science university students

**DOI:** 10.3389/fpsyg.2025.1765344

**Published:** 2026-01-12

**Authors:** Zilong Wang, Ziqi Feng, Huizi Cui, Lingyue Meng, Pengfei Wang, Mengya Lu, Tao Liu, Qiuxia Zhang, Xiangdong Wang

**Affiliations:** 1 School of Physical Education, Jimei University, Xiamen, China; 2 School of Public Foundation, Dalian University of Technology, Panjin, China; 3 School of Physical Education, Soochow University, Suzhou, China; 4 Department of Sports and Leisure, Dongshin University, Naju, Jeollanam-do, Republic of Korea

**Keywords:** mental fatigue, single-leg drop landing, sex differences, biomechanics, sports injury

An incorrect number was provided for the National Social Science Fund of China General Project. The correct number is Grant No. 23BTY120.

The original version of this article has been updated.

